# Synthesis of Sulfoximines and Sulfonimidamides Using Hypervalent Iodine Mediated NH Transfer

**DOI:** 10.3390/molecules28031120

**Published:** 2023-01-22

**Authors:** Renzo Luisi, James A. Bull

**Affiliations:** 1 Department of Pharmacy-Drug Sciences University of Bari “A. Moro” Via E. Orabona 4, 70125 Bari, Italy; 2 Department of Chemistry, Imperial College London, Molecular Sciences Research Hub, London W12 0BZ, UK

**Keywords:** sulfoximines, sulfonimidamides, ammonia, hypervalent iodine, NH transfer, chemoselectivity, drug discovery

## Abstract

The development of NH transfer reactions using hypervalent iodine and simple sources of ammonia has facilitated the synthesis of sulfoximines and sulfonimidamides for applications across the chemical sciences. Perhaps most notably, the methods have been widely applied in medicinal chemistry and in the preparation of biologically active compounds, including in the large-scale preparation of an API intermediate. This review provides an overview of the development of these synthetic methods involving an intermediate iodonitrene since our initial report in 2016 on the conversion of sulfoxides into sulfoximines. This review covers the NH transfer to sulfoxides and sulfinamides, and the simultaneous NH/O transfer to sulfides and sulfenamides to form sulfoximines and sulfonimidamides, respectively. The mechanism of the reactions and the identification of key intermediates are discussed. Developments in the choice of reagents, and in the reaction conditions and setups used are described.

## 1. Introduction

**Figure 1:**
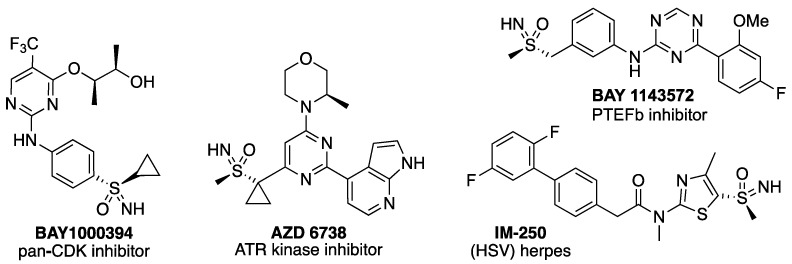
Biologically active sulfoximine-containing compounds.

The sulfoximine and sulfonimidamide functional groups are chemically stable aza-analogues of sulfones and sulfonamides, respectively [1]. The extra N-atom can create a sulfur stereocenter and provide an additional vector for substitution that can be used to tune the properties of a compound. Until relatively recently, these motifs had received little attention outside a few notable applications, as auxiliaries [2], chiral ligands [3,4], and directing groups [5,6]. The development of Bayer’s pan-CDK inhibitor BAY1000394, the first of sulfoximine-containing compounds to enter the clinic [7], thrust sulfoximines into the limelight [8,9], and grabbed the attention of the medicinal and synthetic chemistry communities [10]. Subsequently, sulfoximine-containing compounds entered the clinic, including BAY 1143572, a PTEFb inhibitor, also from Bayer [11,12], and ceralasertib (AZD6738) an ATR kinase inhibitor from AstraZeneca (Figure 1) [13,14,15]. An increasing number of sulfoximine-containing bioactive compounds are now appearing in the literature as the sulfoximine group is both increasingly established and increasingly accessible [16,17], and drug discovery applications were recently reviewed [18,19,20,21]. Sulfonimidamides are less progressed in drug discovery programs, but also offer considerable promise in medicinal and agrochemical applications [22,23,24,25].

Methods for the synthesis of sulfoximines have developed considerably in recent years [26,27,28], with several new approaches for their construction using electrophilic [29,30,31,32,33] and nucleophilic sulfur-containing reagents [34,35,36]. Sulfoximines had traditionally been primarily prepared from sulfoxides through the transfer of a (protected) nitrogen group [28,37,38,39]. Notably, Bolm developed numerous methods for the preparation of sulfoximines using transition metal catalysis with iminoiodinane reagents, using Rh catalysis for the transfer of trifluoroacetamide [40], and silver- [41] and iron-catalysed processes to prepare different N-protected derivatives [42,43]. We contributed to this area through the development of the Rh-catalysed synthesis of sulfoximine carbamates [44,45]. Bolm also demonstrated metal free processes for sulfoximine synthesis from sulfoximines using hypervalent iodine reagents [46,47]. There are relatively fewer, but valuable methods that undertake sulfide imination first followed by oxidation [48,49,50,51,52,53]. 

On the other hand, methods for the direct synthesis of unprotected NH sulfoximines and sulfonimidamides have been less developed. The use of sodium azide in combination with sulfuric acid provided a reactive but hazardous combination for NH transfer, generating HN_3_ as the NH donor [54,55]. This reagent was also investigated in flow for larger-scale application [56]. The strongly acidic conditions lead to the racemisation of enantioenriched sulfoxides. The use of O-mesitylenesulfonylhydroxylamine (MSH) can achieve NH transfer with the retention of the ee of sulfoxides [57,58], but this reagent is explosive [59]. Richards developed an important advancement using Rh catalysis to form NH sulfoximines that used O-(2,4-dinitrophenyl)-hydroxylamine (DPH) as a preactivated amine source [60]. More recently, Bolm used an FeSO_4_/phenanthroline-catalysed process to transfer NH using an arylhydroxylamine derivative [61]. 

This review covers developments since 2016 on the preparation of NH sulfoximines and NH sulfonimidamides using simple sources of ammonia in the form of ammonium salts and hypervalent iodine reagents, following our initial report of an iodonitrene reagent [62]. The review is divided by substrate type, involving either NH transfer to sulfoxides or NH/O transfer to sulfides. The review also covers the preparation of NH sulfonimidamides by a similar set of reagents, through NH and O transfer, from sulfinamides and sulfenamides. These developments have rendered the synthesis of NH sulfoximines and sulfonimidamides more straightforward, with broad functional group compatibility. This review does not intend to cover the subsequent diverse transformations that have been applied using sulfoximine products, which were reviewed elsewhere [26]. The reagent combination has been applied more broadly in processes involving N-transfer that may include other mechanistic steps. This includes the reaction with thiophenols to form sulfonimidates or sulfonamides [63], and the N-transfer to amines [64], N-transfer to form diazirines from amino acids [65,66], and for nitrogen extrusion in molecular editing processes [67,68,69,70].

## 2. Sulfoxides to NH Sulfoximines

**Scheme 1:**
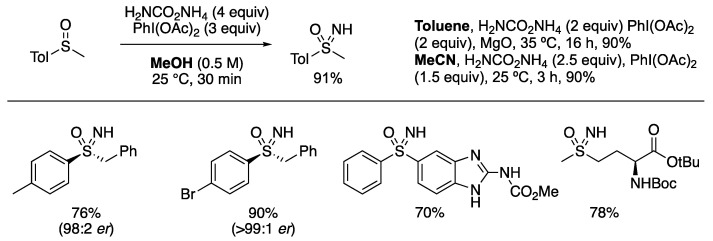
Sulfoximine synthesis by NH transfer to sulfoxides.

In 2016, we (Bull and Luisi) reported the preparation of NH sulfoximines from sulfoxides using bisacetoxyiodobenzene and ammonium carbamate (Scheme 1) [62]. This reagent combination provided an extremely facile approach to form sulfoximine derivatives using convenient and nonhazardous sources of ammonia. The reaction could be run at room temperature in various solvents, including toluene or MeCN, but was fastest in methanol. The reaction displayed a wide substrate scope and excellent functional group tolerance, including for pyridine-like nitrogen atoms and acidic protons. The assessment of the functional group tolerance through the reaction scope and through a robustness screen, as described by Glorius [71], displayed the compatibility for many heterocyclic derivatives and potentially oxidisable functionality such as alkenes without aziridination occurring. More electron-rich heteroarenes such as indoles were not well-tolerated [68]. The reaction proceeds with the retention of any enantiomeric excess in the starting sulfoxide. Subsequently, Luisi developed the method in flow [72].

**Scheme 2:**
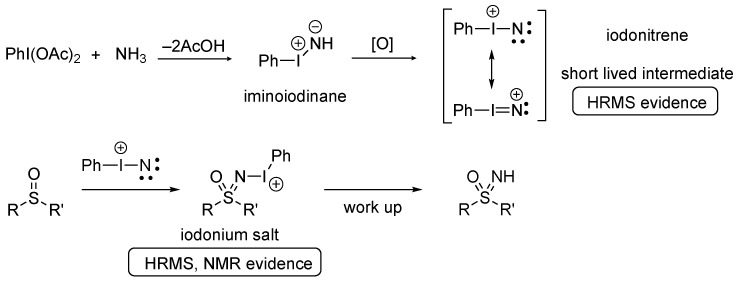
Mechanism for NH transfer to form sulfoximines via an electrophilic iodonitrene.

Studies on the mechanism indicated that the reaction involved the formation of an unprecedented iodonitrene (Scheme 2) [62]. The ammonium carbamate provides a source of ammonia, and this dissolves readily in methanol to provide a methanolic ammonia solution. The use of commercial solution of NH_3_ in MeOH was also successful. Ammonia undergoes condensation with the hypervalent iodine reagent to initially form an iminoiodinane that may react directly with the sulfoxide. More likely is that the iminoiodinane undergoes oxidation to an iodonitrene. Both the iodonitrene and the iminoiodinane are short-lived and not visible by NMR, but they were detected by HRMS performed in flow. 

The electrophilic iodonitrene intermediate is attacked by the sulfoxide as a nucleophile. Monitoring the reaction with in situ NMR indicated that an iodonium salt is directly formed containing the sulfoximine N–I bond, which slowly breaks down or can be worked up to provide the NH sulfoximine [62]. Iodonium salts of this type were subsequently prepared from the isolated sulfoximines and can be applied in further reactions [27,73].

**Scheme 3:**
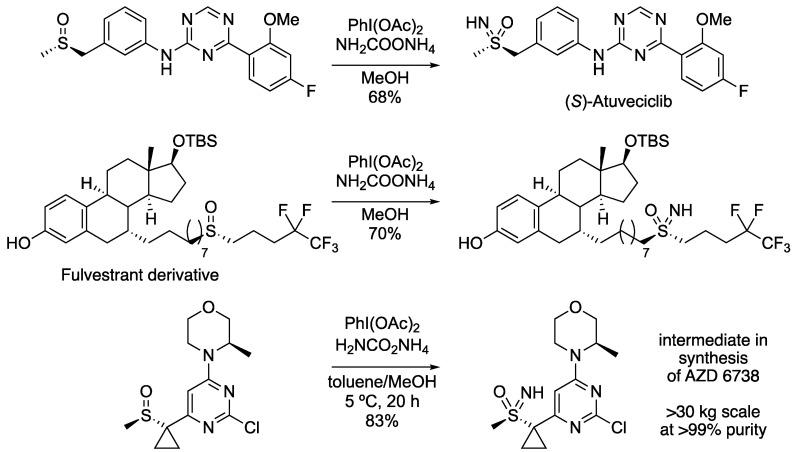
Application of NH transfer methodology to the preparation of pharmaceutical intermediates and derivatives.

Since the discovery of this NH transfer reaction, it has been well-used in the synthesis of sulfoximines, and particularly in the preparation of drug analogues (for example, see Scheme 3). Reboul demonstrated the enantioselective synthesis of (*S*)-atuveciclib [74], and Lücking the preparation of a sulfoximine derivative of fulvestrant [75]. Most notable is the application from AstraZeneca in the preparation of an intermediate for AZD6738 [76]. The sulfoximine-containing intermediate was isolated as the HCl salt on a 30 kg scale in >99% purity and 83% yield. On this large scale, the reaction was performed in a mixture of toluene and methanol to facilitate an aqueous extractive work-up.

## 3. Sulfides to NH Sulfoximines

**Scheme 4:**
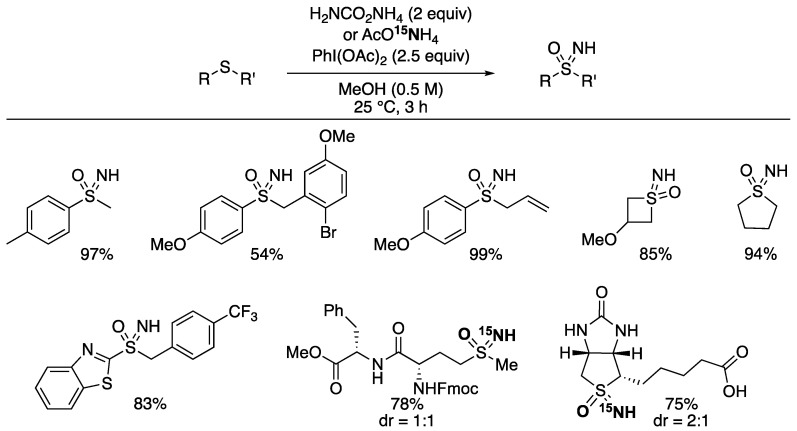
Sulfoximine formation from sulfides using ammonia sources and hypervalent iodine reagent.

In 2017, we (Luisi and Bull) reported the direct conversion of sulfides into sulfoximines using the same set of reagents (Scheme 4) [77]. Similar reports emerged independently from Reboul [78] and Li [79] around the same time. In each case, the one-pot process delivered both NH and O groups to the sulfides to prepare NH sulfoxides in high yields and in convenient procedures. On the sulfides, which are more reactive than the sulfoxide substrates above, we applied 2.5 equiv of PhI(OAc)_2_ as the oxidant, along with 2 equiv of ammonium carbamate as the source of ammonia [77]. The reaction scope was broad and again showed high functional group tolerance of other heteroatoms, protic groups, and oxidatively sensitive sites. The overoxidation of the sulfide into the sulfone was not observed. Under the reaction conditions, both diphenylsulfoxide and diphenylsulfilimine were converted into the sulfoximine products under the reaction conditions, yet only the sulfilimine reacted with the oxidant alone to give the sulfoximine, demonstrating high chemoselectivity. 

Ammonium carbamate could be readily replaced with ammonium acetate. This allowed for the use of the ^15^N labelled ammonium salt and hence the preparation of ^15^N-labelled sulfoximines [77]. This was applied to protected methionine amides and biotin, which further demonstrated the compatibility of the reaction with polar functional groups. 

**Scheme 5:**
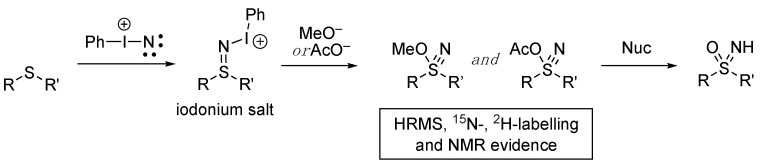
Reboul’s detection of sulfane nitrile intermediates and the proposed mechanism for sulfoximine formation from sulfides.

Reboul’s study was able to identify the presence of intermediates, which gave important further insights into the reaction mechanism [78]. The observation of key sulfane nitrile intermediates using various analytical techniques led to the proposal of a now well-accepted reaction mechanism (Scheme 5). The iodonitrene (see Scheme 2) reacts with the sulfide to initially form an iodonium salt. A formal attack of either acetate or methoxide and the elimination of iodobenzene forms the detected reactive sulfane nitrile intermediates, which contain a triple bond between sulfur and nitrogen and an S–OR bond. The unusual sulfane nitrile group had been previously prepared by Yoshimura, and alkyl derivatives were shown to be reactive alkylating agents to liberate sulfoximines [80,81]. The acidic nature of the reaction mixture causes these intermediates to rapidly break down on the action of various possible nucleophiles (including methanol solvent or the sulfoximine itself) [78].

Li undertook a detailed screen of nitrogen sources. In addition to ammonium carbonate in methanol, ammonium oxalate, ammonium fluoride, ammonium formate, and benzoate also gave good yields [79]. 

**Scheme 6:**
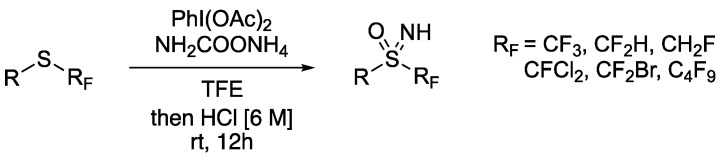
Sulfoximine formation on electron poor fluoroalkyl sulfides using TFE as solvent.

Reboul also developed alternative conditions for the reaction at electron-poor fluoroalkylsulfides (Scheme 6) [82]. Fluorinated derivatives have been of recent interest [83], but due to the mechanistic requirement for a nucleophilic attack on the electrophilic nitrene, these types of substrates were less reactive. Reboul used trifluoroethanol (TFE) to achieve a significant rate enhancement and good yields with otherwise unreactive substrates. The role of the solvent was proposed to form an activated PhI^+^N species through H-bonding. Interestingly, the N–Ac sulfoximine was formed as a significant minor product. To avoid difficult separations, the initial product mixture was treated with HCl (6 M) in MeCN to remove the acetyl group. 

**Scheme 7:**
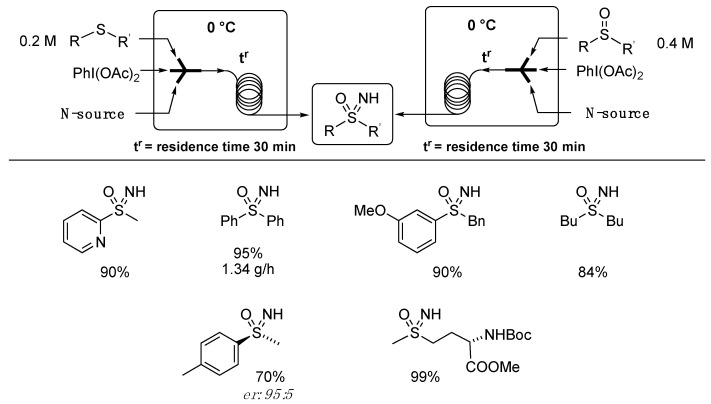
Flow synthesis of NH sulfoximines.

The reaction has seen several further developments in the reagents and reactors used, and an expansion of the reaction scope. Luisi optimized the one pot O- and NH-transfer protocol on sulfides and the NH transfer to sulfoxides under continuous flow conditions. [72]. To avoid the risk of precipitation and clogging of the flow system, ammonium carbamate was replaced with ammonium acetate or aqueous ammonia using a 0.2 M methanolic solution of a sulfide. By using 2 equivalents of PhI(OAc)_2_, 2 equivalents of NH_3_ (aq), and a 15 min residence time at 0 °C, the desired NH sulfoximine was obtained in 95% yield. The use of sulfoxides as a substrate required a 0.4 M concentration, higher concentrations of PhI(OAc)_2_, and N-source, and 30 min of residence time (Scheme 7). In comparison to a batch approach, the use of the flow technology allowed for reducing the equivalents of both oxidant and source of ammonia. As with the batch protocol, the flow method performed very well in terms of yields and functional group tolerance. The continuous flow synthesis of biologically relevant methionine sulfoximine (MTO) and enantioenriched sulfoximine were tested, and long run continuous flow synthesis returned a productivity of 1.34 g/h for phenyl methyl sulfoximine.

**Scheme 8:**
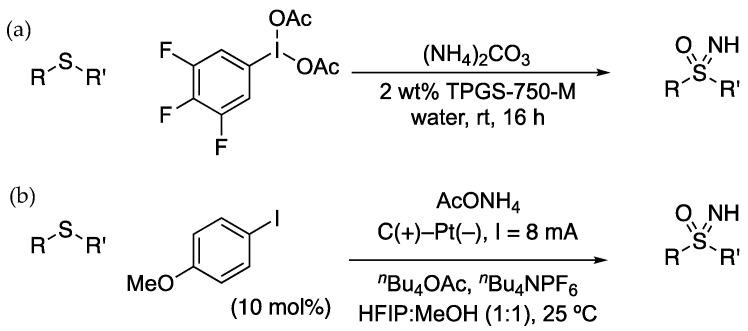
(**a**) Sulfoximine formation in water using a recyclable hypervalent iodine reagent. (**b**) Electrochemical sulfoximine formation, with catalytic aryl iodide.

Zheng and Xu reported the NH/O transfer reaction in water using a recyclable iodine (III) reagent (Scheme 8a) [84]. Several arenes were examined on the aryl-I(III) reagents, and the 3,4,5-trifluorophenyl derivative gave the best outcome in both yield and recoverability. Reactions were run under micellar conditions to improve the solubility of the oxidant in water by the addition of a surfactant (TPGS-750-M). The iodide was then recovered after the reaction by extraction into heptane in an aqueous workup. An aryl iodide has also been used catalytically, along with an electrochemical oxidation to convert sulfides to sulfoximines (Scheme 8b). Kong, Wang, and Xu developed the anodic oxidation of trimethoxyiodobenzene to the corresponding I(III) reagent, which promoted the overall conversion of sulfides into sulfoximines [85]. 

A description of all applications of these conditions is outside of the scope of this review [26,86]. However, notable extensions have displayed interesting selectivity and functional group tolerance. Bräse applied the reaction to bicyclo[1.1.1]pentyl (BCP) sulfides in the synthesis of BCP sulfoximines [87]. Zhang and Chen reported the tandem formation of sulfoximines from sulfides and intramolecular C–H amination, where (NH_4_)_3_PO_4_·3H_2_O was used as the ammonia source, and the reaction could be run at RT under air in methanol or in DMF [88]. With Rollin, we reported the diastereoselective preparation of glycosylsulfoximines achieving dr of up to 95:5 [89]. With Craven and Armstrong, we reported the preparation of vinyl sulfoximines as possible chiral covalent warheads [90]. Several approaches to vinyl sulfoximines were developed, including a short hydrothiolation of alkynes and NH/O transfer sequence to form the vinyl sulfoximines.

**Scheme 9:**
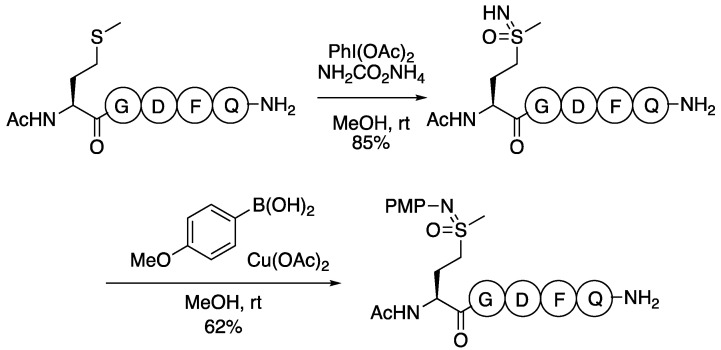
Sulfoximine formation and functionalisation on methionine residues in poly peptides.

Very recently, Ball applied the NH/O transfer method to the functionalisation of methionine in polypeptides, and subsequent N-arylation to enable protein labelling (Scheme 9) [91]. The preparation of methionine sulfoximines had been demonstrated previously on small amino acid derivatives. In this recent work, methionine residues in polypeptides are selectively converted to sulfoximines. Slightly adjusted conditions used a larger excess of ammonium carbamate (20 equiv). Subsequently, a chemoselective sulfoximine N-arylation was demonstrated using copper acetate and arylboronic acids [92]. The reaction was demonstrated on up to 13-mer peptides in methanol or methanol water combinations, with yields determined by HPLC. Tyrosine and tryptophan residues were well-tolerated, as were other nucleophilic residues, though cysteine was oxidised. Together, these present a potential approach for peptide functionalisation and bioconjugation.

**Scheme 10:**
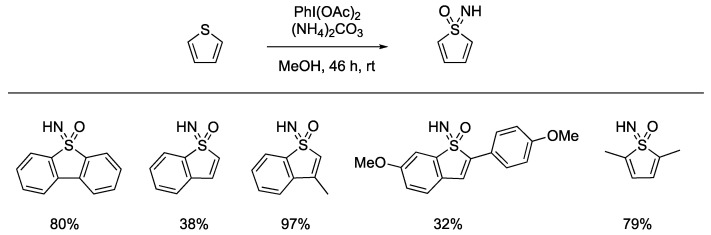
Thiophene NH-sulfoximine formation.

Bolm reported the preparation of thiophene sulfoximines as analogues of thiophene sulfones that are used in thiophene-based materials. Using NH/O transfer on this different substrate class of thiophene derivatives used 5 equivalents of PhI(Oac)_2_ and 3 equiv of ammonium carbonate as the ammonia source. Benzothiphenes were successful in this reaction, as was 2,5-dimethylthiophenes, and the substituents affected reaction yield (Scheme 10).

## 4. Sulfinamides to NH Sulfonimidamides

Sulfonimidamides, the aza-analogue of sulfonamides, have received relatively little synthetic attention, but have seen several developments in the last 10 years [22,23,24,25]. Approaches have been reported to sulfonimidamides primarily through sulfonyl halide reagents [29,32,93,94] and the N-transfer to sulfinamides [95,96].

**Scheme 11:**
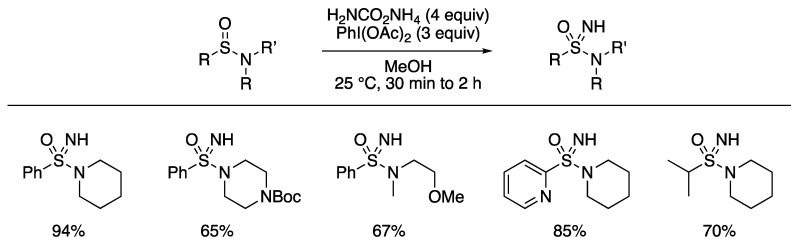
Lücking and Stockman’s NH transfer to sulfinamides to form NH sulfonimidamides.

In 2017, Lücking and Stockman developed a facile NH transfer reaction to form NH sulfonimidamides from sulfinamides using the combination of ammonium carbamate and PhI(Oac)_2_ [97]. The application of the conditions, reported for the conversion of sulfoxides into sulfoximines [62], gave good yields for tertiary sulfinamides (Scheme 11). The reaction was demonstrated with a range of cyclic and acyclic nitrogen substituents, and with variation in the sulfinamide carbon substituent across aryl and alkyl derivatives.

Using primary sulfinamides gave the corresponding sulfonimidate. By comparison, primary sulfinamides in the presence of hypervalent iodine reagents were reported by Malacria to form sulfonimidates in the presence of alcohols [98], as do secondary N-arylsulfinamides [99,100]. A mixture of side products was obtained with secondary sulfinamides, including the sulfonamide and sulfonimidate. Lücking and Stockman also developed a broad range of reactions for the N-functionalisation of the NH sulfonimidamides [101].

**Scheme 12:**
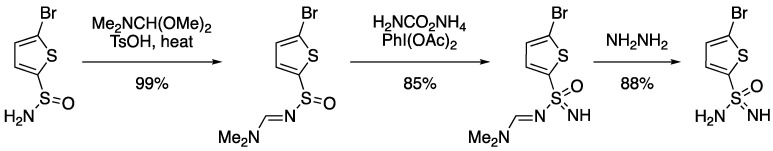
NH transfer on sulfinylamidines to form primary sulfonimidamides.

Lu reported NH transfer on sulfinylamidines, which acted as a protecting group for primary sulfinamides [102]. An example on a thiophene derivative gave the primary NH sulfonimidamide (Scheme 12), which could be further derivatised. The use of enantioenriched *tert*-butylsulfinamidine with the NH transfer protocol gave a racemic sulfinylamidine derivative due to intramolecular migration of the amidine group.

## 5. Sulfenamides to NH Sulfonimidamides

**Scheme 13:**
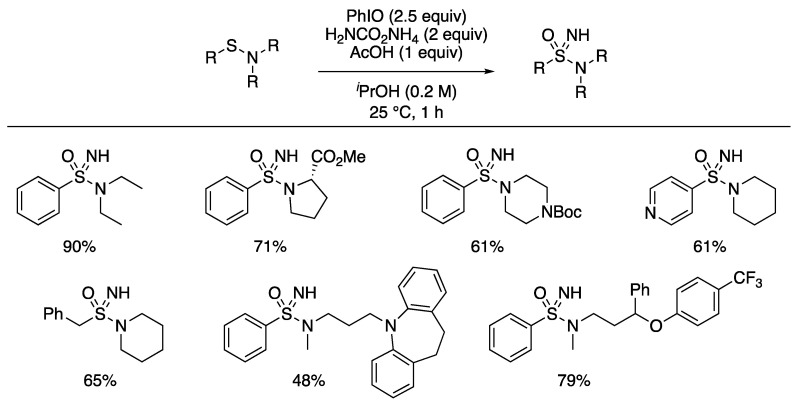
Synthesis of sulfonimidamides from sulfenamides by NH transfer using iodosylbenzene and ammonium carbamate in *i*PrOH.

In 2019, we (Bull and Luisi) reported the preparation of NH sulfonimidamides through a one-pot NH and O transfer reaction from sulfenamides [103]. Sulfenamides were prepared from amines and disulfides, using silver nitrate, under conditions reported by Davis [104]. Treating sulfenamides with conditions related to those reported for the preparation of sulfides from sulfoximines (PhI(Oac)_2_ and ammonium carbamate in methanol) gave the sulfonimidamide as the major product. However, the acidic conditions also gave various side products. As such, the reaction was reoptimized to use iodosylbenzene in *i*PrOH with the addition of 1 equivalent of acetic acid (Scheme 13).

**Scheme 14:**
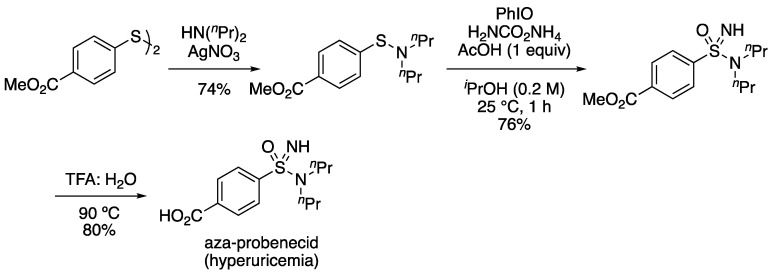
Preparation of aza-analogue of probenecid.

The reaction gave high yields with tertiary sulfenamides. Enantiopure amine derivatives retained the er in the amine component. Cyclic and acyclic amine derivatives and alkyl and aryl groups on sulfur were tolerated. Furthermore, the process was applied to the formation of sulfonimidamides from drug compounds containing secondary amines, desipramine and fluoxetine.). Secondary sulfenamides derived from primary amines gave low yields of the sulfonimidamides and more complex reaction outcomes. An aza-analogue of sulfonamide-containing drug probenecid, a treatment for gout, was also prepared in a short sequence (Scheme 14). The sulfenamide was formed from the corresponding diaryldisulfide and di-*n*-propylamine. Treatment with the revised reaction conditions gave the NH sulfonimidamide. Ester hydrolysis under acidic conditions gave the aza-probenacid. Additionally, the ester derivative was further functionalized through the sulfonimidamide nitrogen by alkylation, acylation and arylation.

**Scheme 15:**
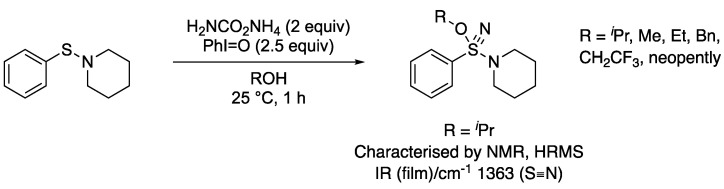
Alkoxy-amino-λ^6^-sulfanenitriles formed in the absence of acetic acid.

The mechanism of the reaction was investigated and shown to proceed via the formation of new amino-alkoxy-sulfane nitrile derivatives. In the absence of the acetic acid additive, new sulfane nitrile derivatives were formed as the major product and could be fully characterized (Scheme 15). Performing the reaction in other alcohols, including methanol, benzyl alcohol, and trifluoroethanol, formed the corresponding substituted sulfane nitriles.

**Scheme 16:**
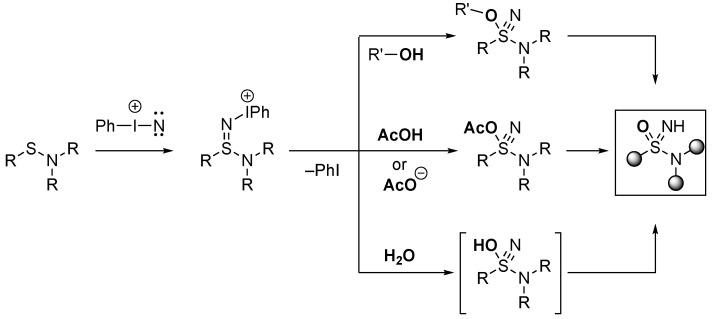
Mechanism for the formation of sulfonimidamides from with alternative sources of the oxygen atom.

A series of mechanistic investigations were undertaken that indicated that the alkoxysulfane nitriles was one of the possible routes to the sulfoximine products [103]. These derivatives were stable in alcoholic solvent, but in the presence of mild acids (such as acetic acid, thiols, phenols), they readily break down to form the sulfonimidamide, acting as an alylating agent. In addition to the alcohols providing the oxygen atom, small quantities of water were incorporated, and the acetate of the acetic acid could also act as the source of the O atom in the product. The acetate-bearing intermediate was unstable and was hydrolysed directly by the action of the solvent to form esters and generate the sulfonimidamide. Each of these routes led to the sulfonimidamide under the optimised mildly acidic conditions (Scheme 16).

**Scheme 17:**
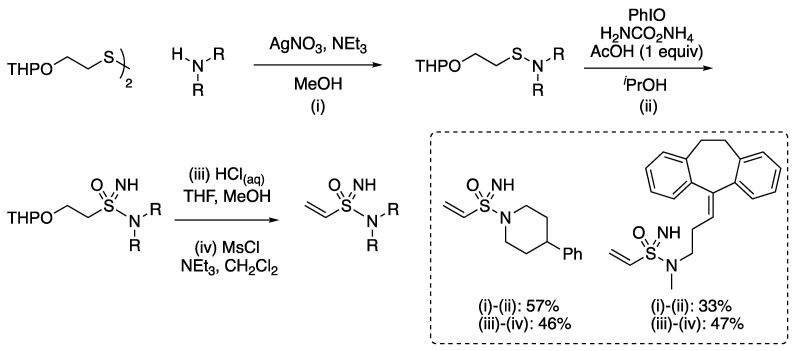
Synthesis of vinyl sulfonimidamides.

With Craven and Armstrong, we demonstrated the sequence for the preparation of vinyl sulfonimidamides as a possible alternative to acylamides in covalent probes, providing a chiral warhead [90]. Using a disulfide derived from protected mercaptoethanol gave sulfenamides with secondary amines (Scheme 17). The addition of Et_3_N gave improved yields in the sulfenamide formation through buffering the acidity of the reaction mixture. The application of the modified conditions for NH/O transfer gave a sulfonimidamide that could be converted into the vinyl derivative by THP deprotection and the elimination of the alcohol by mesylation under mild basic conditions.

## 6. Conclusions

The combination of hypervalent iodine (III) reagents and simple sources of ammonia in alcohol solvents are powerful reagents for NH transfer. In the presence of sulfoxides, sulfoximines are formed with excellent yields, a tolerance of polar, protic, and basic functionality, and the retention of er from the sulfoxide. Various ammonium salts have been applied, the reaction is tolerant of several solvents and solvent mixtures, and is scalable. The reaction is understood to involve the formation of an iodonitrene. Using sulfides, the same reagent combination also lead to the formation of sulfoxmines with the transfer of an O atom and an NH group. This provides a short and convenient route to sulfoximines, and the reaction again displayed broad functional group tolerance. The reaction has also been performed in flow, using electrochemical oxidation, and in water. It has been applied to methionine-containing polypeptides and to thiophenes. Similarly, sulfinamides and sulfenamides were converted into sulfonimidamides that provide aza-analogues of sulfonimidamides. Along with broad developments in the field, this reagent combination has helped in making these aza-S(VI) motifs more readily available, and facilitated their application in drug discovery programs.

## Data Availability

Not applicable.
